# Transcranial alternating current stimulation (tACS) above individual peak alpha frequency (PAF) does not shift PAF or experimental pain sensitivity: an exploratory study

**DOI:** 10.3389/fnhum.2026.1851160

**Published:** 2026-07-07

**Authors:** Irit Weissman-Fogel, Reem Sheban, Alon Sinai, Yelena Granovsky, Roi Treister

**Affiliations:** 1 Sensory Neuroscience and Pain Laboratory, Physical Therapy Department, Faculty of Social Welfare and Health Sciences, University of Haifa, Haifa, Israel; 2 Department of Neurosurgery, Rambam Health Care Center, Haifa, Israel; 3 Department of Neurology, Rambam Health Care Center, Haifa, Israel; 4 The Laboratory of Clinical Neurophysiology, Bruce Rappaport Faculty of Medicine, Technion-Israel Institute of Technology, Haifa, Israel; 5 The Clinical Pain Innovation Laboratory, The Cheryl Spencer Department of Nursing, Faculty of Social Welfare and Health Sciences, University of Haifa, Haifa, Israel

**Keywords:** experimental pain, neuromodulation, pain intensity, peak alpha frequency, transcranial alternating current stimulation

## Abstract

**Introduction:**

Peak alpha frequency (PAF) is negatively associated with experimental pain sensitivity. Transcranial alternating current stimulation (tACS) enables shifting neural frequency to modulate sensory perception, yet it has not been tested in pain perception. We hypothesized that tACS application at 1 Hz above the individual PAF (iPAF) would increase PAF and reduce experimental pain ratings.

**Methods:**

This is a double-blind, sham-controlled, crossover, exploratory study with 15 healthy participants who completed 2 sessions; 20 min of tACS was applied to the somatosensory cortex at 1 Hz above the iPAF or sham stimulation (Intervention), administered in random order. Pain reports (4 time points) in response to a tonic heat pain (THP) stimulus were recorded before and after tACS. Five-minute electroencephalography was recorded in 4 conditions: baseline (iPAF identification), after THP stimulus, after tACS to test its effect on iPAF, and after another THP stimulus to test pain effects on iPAF following tACS. This paradigm examines associations between iPAF and pain, and the effects of pain and tACS on iPAF.

**Results:**

There was no effect of real vs. sham tACS on iPAF, with a non-significant Condition × Intervention interaction and a small effect size (partial *η*^2^ 0.01 and 0.03). Pain intensity increased across rating time points (*p* < 0.001), but tACS had no effects.

**Discussion:**

In this small-sample preliminary study, a single tACS session did not affect iPAF or pain ratings in response to THP stimulus. The current study may help shape methodologies for future pain studies investigating the effects of tACS on PAF and pain.

## Introduction

Pain perception is based on complex temporal–spectral patterns of brain activity. Neuronal oscillations associated with pain range from fluctuations below 0.1 Hz via theta (4–7 Hz), alpha (8–12 Hz), and beta (13–29 Hz) to gamma (30–100 Hz) oscillations (Ploner et al., 2017). Specifically, during experimental prolonged pain, there is a decrease in alpha activity and a shift of peak alpha frequency (PAF) toward lower frequencies in the somatosensory cortex (Ploner et al., 2006; Peng et al., 2014; Nickel et al., 2020). Further, a slower individual PAF (iPAF) recorded at a resting state is associated with higher pain ratings during a prolonged experimental pain condition (Furman et al., 2018, 2019, 2020) and individuals whose PAF slowed during the prolonged pain reported greater pain intensity (Furman et al., 2018). Yet, these findings did not generalize to brief experimental pain stimuli (May et al., 2025). Therefore, modulating PAF in the somatosensory cortex may alter an individual’s pain sensitivity to prolonged experimental pain.

One method that interferes with brain oscillations is transcranial alternating current stimulation (tACS) (Hohn et al., 2019). tACS is based on neural entrainment, which involves modulating endogenous brain oscillations at a specific frequency to synchronize with external rhythmic stimulation. In this way, tACS can facilitate neuronal activity at a specific frequency, thereby altering brain function and related behaviors (Polanía et al., 2018; Vosskuhl et al., 2018; Tavakoli and Yun, 2017). Few studies have investigated the effects of tACS applied to the somatosensory cortex at alpha frequency (10 Hz) on experimental and clinical pain (Fathi et al., 2026). In two studies, tACS was applied during either a brief pressure pain stimulus (Arendsen et al., 2018) or prolonged experimental pain (May et al., 2021), compared with a sham condition. In the former (Arendsen et al., 2018), tACS reduced pain ratings compared to sham only when the intensity of the upcoming stimulus was uncertain, suggesting that tACS modulates pain anticipation and perception in a context-dependent manner (Li et al., 2025). In the latter, tACS did not affect pain sensitivity (May et al., 2021), as was further revealed by a study that applied tACS at the individual PAF (iPAF) (Fathi et al., 2025). In another study, 40 min of tACS at 10 Hz was applied in a randomized, double-blind, sham-controlled, crossover design in patients with chronic low back pain. A significant enhancement of alpha activity was identified following real tACS vs. sham, which was correlated with a reduction in clinical pain severity and perceived disability (Ahn et al., 2019). These studies provide some evidence of a causal link between alpha frequency and pain sensitivity, depending on the type of pain stimulus (May et al., 2025; Liberati, 2025).

tACS can also be applied at a frequency slightly above or below the neural frequency to shift the endogenous frequency to the tACS frequency to alter behavior or perception (Vosskuhl et al., 2018). For example, tACS above the individual gamma frequency, known to be involved in the temporal resolution feature of auditory processing, increased frequency towards the tACS frequency, and improved auditory resolution (Baltus et al., 2018). In another study, tACS applied below and above PAF, enlarged and shrunk, respectively the temporal window for perceiving a sound-induced double-flash illusion (Cecere et al., 2015) and modified the duration of perceived visual stimulation, such that progressively “faster” alpha stimulation led to longer perceived intervals (Mioni et al., 2020). These behavioral after-effects suggest neural plasticity as an underlying mechanism (Tavakoli and Yun, 2017).

To summarize, the literature suggests an association between the iPAF and pain sensitivity. Further, a single session of tACS can shift neural oscillations toward its frequency, thereby modulating perceptions (visual and auditory). We therefore hypothesized that a single session of tACS applied above the iPAF would shift PAF toward faster frequency and reduce experimental pain sensitivity. Here, we focus on changes that persist after stimulation ends. Given the exploratory nature of this first study on shifting iPAF to the tACS frequency, the sample size was determined based on an acceptable number of participants for a pilot study. Understanding such aftereffects in healthy participants is an essential step toward developing these techniques into potentially useful clinical tools for treating specific patient groups.

## Methods

This is a double-blind, sham-controlled, crossover, exploratory study. The study was approved by the Institutional Review Board (IRB) of the Faculty of Social Welfare and Health Sciences at the University of Haifa (approval no. 405/20). The subjects were recruited through an advertisement posted on social networks in various groups and via the snowball method.

### Participants

*Inclusion criteria:* Healthy subjects between 18 and 65 years, right-handedness, and ability to sign an informed consent form and understand the experiment instructions. *Exclusion criteria:* acute or chronic pain conditions, metabolic, cardiovascular, neurological, psychiatric disorders, attention deficit disorders, regularly taking medication (except for birth control pills), tinnitus and dizziness, pregnant/lactating women, and drug users. Contraindications to the use of electric current stimulation: epilepsy, pacemaker, migraine, severe headaches, metallic body or metallic implants, and wounds. Participants were asked to avoid drinking alcohol 48 h before the experiment.

Participants whose PAF was >11 Hz, as determined by the electroencephalogram (EEG) conducted at the beginning of the experiment, were excluded to avoid shifting the iPAF into beta waves, which may involve different neurophysiological mechanisms.

### Study procedure

Upon arriving at the lab, participants received a detailed oral and written explanation of the experiment, signed an informed consent form, and completed demographic and general health questionnaires. Following this, a familiarization with the heat pain stimulus and pain rating scale was conducted. An EEG cap was placed on the participant’s head, and a recording of brain activity was done to identify the individual PAF at rest.

The study consisted of two sessions, separated by at least 1 week. tACS was applied at 1 Hz above the individual PAF or a sham tACS, in a random order. The sessions were held in the morning in a room maintained at a controlled 23 °C.

**Figure 1:**
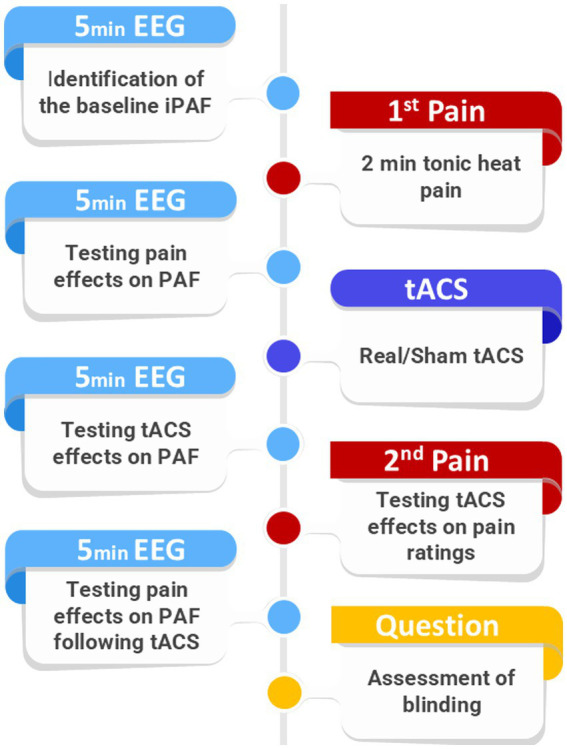
The study flow chart. EEG, electroencephalogram; tACS, transcranial alternating current stimulation; iPAF, individual peak alpha frequency.

Each session included 7 procedures (Figure 1) as follows: (1) 5 min of EEG recording at resting state in order to identify the iPAF; (2) 120 s of contact tonic heat pain (THP) stimulation at 46.5 °C. Each participant was asked to rate the intensity of the pain using the Numerical Rating Scale (NRS) after 30, 60, 90, 120 s; (3) 5 min of EEG recording in order to evaluate the effect of THP on the PAF; (4) 20 min of tACS at 1 Hz above the iPAF or sham tACS; (5) 5 min of EEG recording in order to identify changes in PAF attributed to the tACS stimulation; (6) 120 s THP to test if tACS affected THP ratings; (7) 5 min of EEG recording in order to exam if changes in pain experience is linked with changes in PAF. This enables an exploration of the after-effects of tACS outlasting the stimulation, taking into consideration the effects of continuous pain on PAF (Furman et al., 2020; De Martino et al., 2021; Valentini et al., 2022) (Figure 1).

### Experimental pain

#### Familiarization with heat pain and pain ratings

A Peltier thermode [Thermal Sensory Analyzer (TSA2), Medoc, Ramat Yishai, Israel] 30 × 30 mm in size was attached to the volar surface of the forearm of the dominant hand. Each participant received 3 heat stimuli with a 15-s inter-stimulus interval. The baseline temperature was 32 °C and the increased rate was 2 °C/s rate until reaching the target temperature of 45 °C, 46 °C and 47 °C, in a random order, and remaining fixed for 7 s. Participants were requested to rate pain intensity induced by each stimulus using the NRS ranging from 0 to 100 (0 denotes ‘no pain’ to 100 maximum pain imaginable), just before the stimulus returned to baseline.

#### Tonic heat pain (THP)

The TSA2 thermode was attached to the volar surface of the left forearm. The temperature was increased at a rate of 2 °C/s from 32 °C to 46.5 °C, then held constant for 120 s. This temperature was chosen based on previous studies showing that it induces moderate pain intensity (Tousignant-Laflamme et al., 2008). Participants were asked to rate pain intensity on the 0–100 NRS at 4 time points: 30, 60, 90, and 120 s. The average of the 4 pain ratings was used to test the tACS effects on pain ratings.

### tACS

The tACS current was delivered using a Low-Intensity transcranial stimulator (tES) device (Soterix medical, Woodbridge, New Jersey). The device has a sham option that increases the current to the desired intensity (1.0 mA peak-to-peak) and turns off after 30 s. The subject’s experience is similar to that in the active condition.

The tACS current was delivered through 2 electrodes, which were covered with sponges (5 × 5 cm), immersed in saline which were placed on the scalp in the area of the somatosensory cortex CP3 and CP4, in line with recent recommendations (Bikson et al., 2018). The electrodes were placed under the EEG cap to prevent them from being displaced. The frequency was adjusted for each individual and set about 1 Hz above the iPAF measured in the somatosensory cortex via EEG. The 1 Hz above iPAF was chosen because previous literature has demonstrated 0.5 Hz differences in PAF, with significant differences in individual pain scores (Furman et al., 2018; Nir et al., 2010). We therefore tried to modify the PAF in a small yet meaningful step. During the real stimulation, the current increased for 30 s, ramping up to 1 mA peak-to-peak, then remained at 1 mA for 20 min, and decreased for 30 s, ramping down. We chose 1 mA, which is in the lower range of recommended currents to support blinding.

### EEG recordings

Brain activity at rest was recorded with an EEG system (EasyCap, Brain Products, Munich, Germany) using 13 passive electrodes. The electrodes were placed on the scalp according to the International 10–20 system and were located at Fp1, Fp2, F3, F4, C3, C4, P3, P4, O1, O2, Fz, Cz, Pz. Participants were asked to stay in a relaxed, wakeful state, keeping their eyes open, gazing on a fixation cross. The sampling frequency was 512 Hz, and the bandpass filter was set to 0.1–100 Hz. Impedances were kept below 5 kOhms, and a notch filter of 50 Hz to reduce electrical interference. The recordings were done relative to an averaged reference.

### EEG analysis

The data were analyzed offline using Analyzer Software (Brain Products GmbH, Munich, Germany). Data from the C3 and C4 recording electrodes were analyzed. Each 5-min EEG recording was segmented into 300 1-s segments. All segments were visually inspected for artifacts (electrooculographic or muscular) and excluded if their amplitude exceeded 50 mV. The total number of segments analyzed for each condition ranged from 250 to 300. Power spectral analysis was computed by averaging the fast Fourier transform power spectra of the 1-s data windows from all artifact-free segments. The alpha range was defined as 8–12 Hz. PAF was obtained for each electrode by measuring the exact frequency (with 0.1 Hz resolution) of the peak in the maximal power spectrum between 8 and 12 Hz.

### Assessment of blinding

We aimed at a double-blind design and therefore each session was conducted by a main experimenter who interacted with the participants and both were unaware of the stimulation protocol, i.e., real/sham. Another research assistant (not the one interacting with the subjects) operated the tACS to induce real or sham stimulation based on a randomization list that was prepared in advance and was visible only to him. At the end of the experiment, quality of blinding was assessed by the question “Out of the two sessions you went through, in one session you were not given an electrical stimulation through the electrodes attached to your head. Can you guess in which of the sessions you did not receive stimulation?”

### Statistical analysis

Statistical analyses were conducted using SAS version 9.4 for Windows (Cary, North Carolina, U.S.). To assess potential differences in demographic variables and blinding efficacy between participants allocated to intervention sequence (first session-second session: sham-real; real-sham), an unpaired *t*-test was used for continuous variables and Fisher’s exact test for categorical variables.

To examine the impact of the tACS on PAF outcome in C3 and C4 (separately), we employed a 4 (Condition: Resting state; After 1st pain stimulation; After tACS; After 2nd pain stimulation) * 2 (Intervention: real tACS; sham tACS) mixed-model repeated-measures analysis of variance (ANOVA), controlling for the intervention sequence (sham-real; real-sham), accounting for the fact that subjects are nested in a sequence.

To assess the effects of the intervention (real tACS; sham tACS) on pain intensity during the THP stimulation, a 2 (Intervention: real tACS; sham tACS) * 2 (Time: before and after tACS) * 4 (Ratings: 30, 60, 90, and 120 s during the THP stimulation) a mixed-model ANOVA was applied, controlling for intervention sequence (sham-real; real-sham), accounting for the fact that subjects are nested in sequence.

The significance level for all analyses was set at *α* = 0.05. In the event of a significant interaction or main effect, Bonferroni-corrected pairwise comparisons were planned for *post-hoc* exploration. Partial eta-squared (*ηp*^2^) values are reported as measures of effect size.

A formal *a priori* power analysis was not conducted for this study. The exploratory nature of this research primarily drove this decision, as it represents the first investigation aimed at shifting the iPAF to the tACS’ frequency and assessing its effects on experimental pain sensitivity. Consequently, there was no existing empirical literature or prior data from which to derive reliable effect size estimates necessary for a robust power calculation. Instead, the sample size for this study was determined based on an acceptable number of participants in a pilot study (Julious, 2005).

## Results

### Participants

Twenty healthy participants signed informed consent and arrived at the laboratory for the first session. Five participants were excluded because their alpha frequency was above the predetermined value of 11 Hz. The other 15 participants completed the two sessions and were included in the data analyses.

Most participants were women (*n* = 13) with an average (±SD) age of 23.2 ± 6.9 years, ranging from 18 to 46 years old. There were no significant differences in these demographic variables between the two stimulation sequences (real-sham; sham-real).

### The effects of pain and tACS on PAF

**Figure 2:**
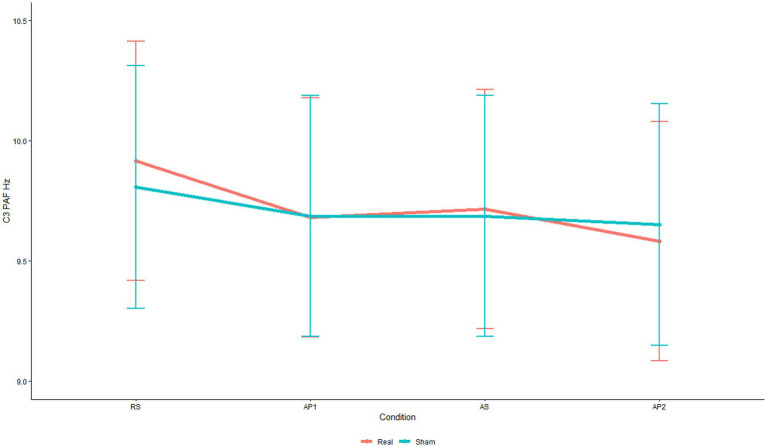
PAF at C3 as a function of tACS (real, sham) and condition. Peak alpha frequency at C3 in the real and sham tACS along the 4 study conditions. PAF, peak alpha frequency; RS, resting state; AP1, after 1st pain stimulation before tACS; AS, after tACS; AP2, after 2nd pain stimulation following tACS.

**Figure 3:**
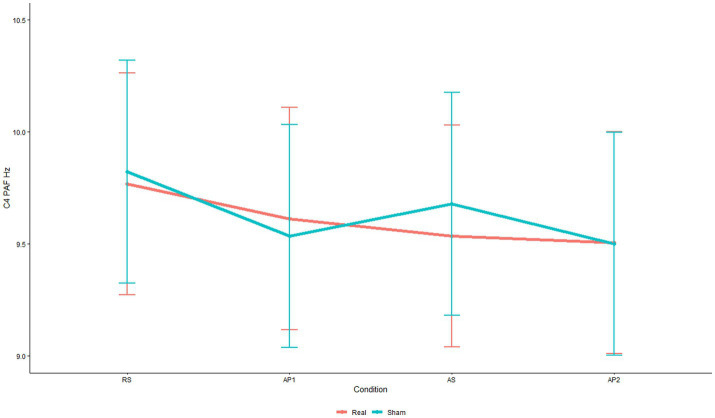
PAF at C4 as a function of tACS (real vs. sham) and condition. Peak alpha frequency at C4 in the real and sham tACS along the 4 study conditions PAF, peak alpha frequency; RS, resting state; AP1, after 1st pain stimulation before tACS; AS, after tACS stimulation; AP2, after 2nd pain stimulation following tACS.

The changes in PAF (at resting state) at C3 and C4 across conditions (resting state, after 1st pain stimulation before tACS, after tACS stimulation, after 2nd pain stimulation following tACS) and interventions (real tACS, sham tACS) are presented in Table 1, Figures 2, 3.

**Table 1:** PAF at C3 and C4 as a function of intervention (real and sham tACS) and condition.

	Average	SD	Minimum	Maximum
Real tACS				
*PAF at C3 (Hz)*				
Resting state	9.9	0.9	8.5	11.5
After 1st pain stimulation	9.6	0.8	8.5	11.5
After tACS stimulation	9.7	0.8	8.5	11.0
After 2nd pain stimulation	9.5	0.9	8.0	11.5
*PAF at C4 (Hz)*				
Resting state	9.7	0.9	8.5	11.0
After 1st pain stimulation	9.5	0.8	8.5	11.0
After tACS stimulation	9.5	0.9	8.5	11.0
After 2nd pain stimulation	9.4	1.0	8.0	11.0
Sham tACS				
*PAF at C3 (Hz)*				
Resting state	9.7	0.9	8.5	11.5
After 1st pain stimulation	9.6	1.0	8.5	11.5
After tACS stimulation	9.6	0.9	8.5	11.5
After 2nd pain stimulation	9.6	0.9	8.5	11.5
*PAF at C4 (Hz)*				
Resting state	9.8	0.7	8.5	10.5
After 1st pain stimulation	9.5	0.8	8.5	10.5
After tACS stimulation	9.6	0.8	8.5	10.5
After 2nd pain stimulation	9.4	1.0	8.0	10.5

PAF, peak alpha frequency; SD, standard deviation; values represent mean ± SD of resting-state PAF measurements.

A mixed-design ANOVA revealed a significant main effect of condition on PAF at C3, *F*(3,86) = 3.00, *p* = 0.034, *ηp*^2^ = 0.09, indicating a significant change in PAF between conditions across interventions. *Post-hoc* pairwise comparisons showed a significant decrease in PAF after the 2nd pain stimulation following tACS compared with the resting state (*P*adj = 0.031). No significant effect was found for the intervention order, and no interaction between condition x intervention was found (Table 2).

**Table 2:** The effects of the intervention (real and sham tACS) and condition on PAF.

	*F*-value	*p*-value	Effect size^a^
C3			
Condition	3.00	**0.034**	0.09
Intervention	0.01	0.90	9.37e−04
Intervention order	0.50	0.48	0.03
Condition × intervention	0.38	0.76	0.01
C4			
Condition	5.35	**0.002**	0.16
Intervention	0.00	0.96	7.52e−05
Intervention order	0.62	0.43	0.02
Condition × intervention	0.74	0.52	0.03

a Partial *η*^2^, effect size, according to Cohen (1988) considered 0.01 partial eta squared effect as small, 0.06 considered medium and 0.14 considered large. Significant *p*-values are in bold font.

A mixed-design ANOVA also revealed a significant main effect of condition on PAF at C4, *F*(3,85) = 5.35, *p* = 0.002, *ηp*^2^ = 0.16, indicating a significant change in PAF between conditions across interventions. *Post-hoc* pairwise comparisons showed a significant decrease in PAF after the 1st pain stimulation (*p*adj = 0.032) and the 2nd pain stimulation applied following tACS (*p*adj = 0.002), each compared with the resting state. No significant effect was found for the Intervention order, and no interaction between Condition × Intervention was found (Table 2).

A sensitivity analysis conducted in G*power indicated that the study was powered to detect a Condition × Intervention interaction with *f* = 0.218 (equivalent to partial *η*^2^ = 0.045) or larger. This suggests that the study was capable of detecting small-to-medium interaction effects.

### Effects of tACS (real vs. sham) on pain intensity

**Figure 4:**
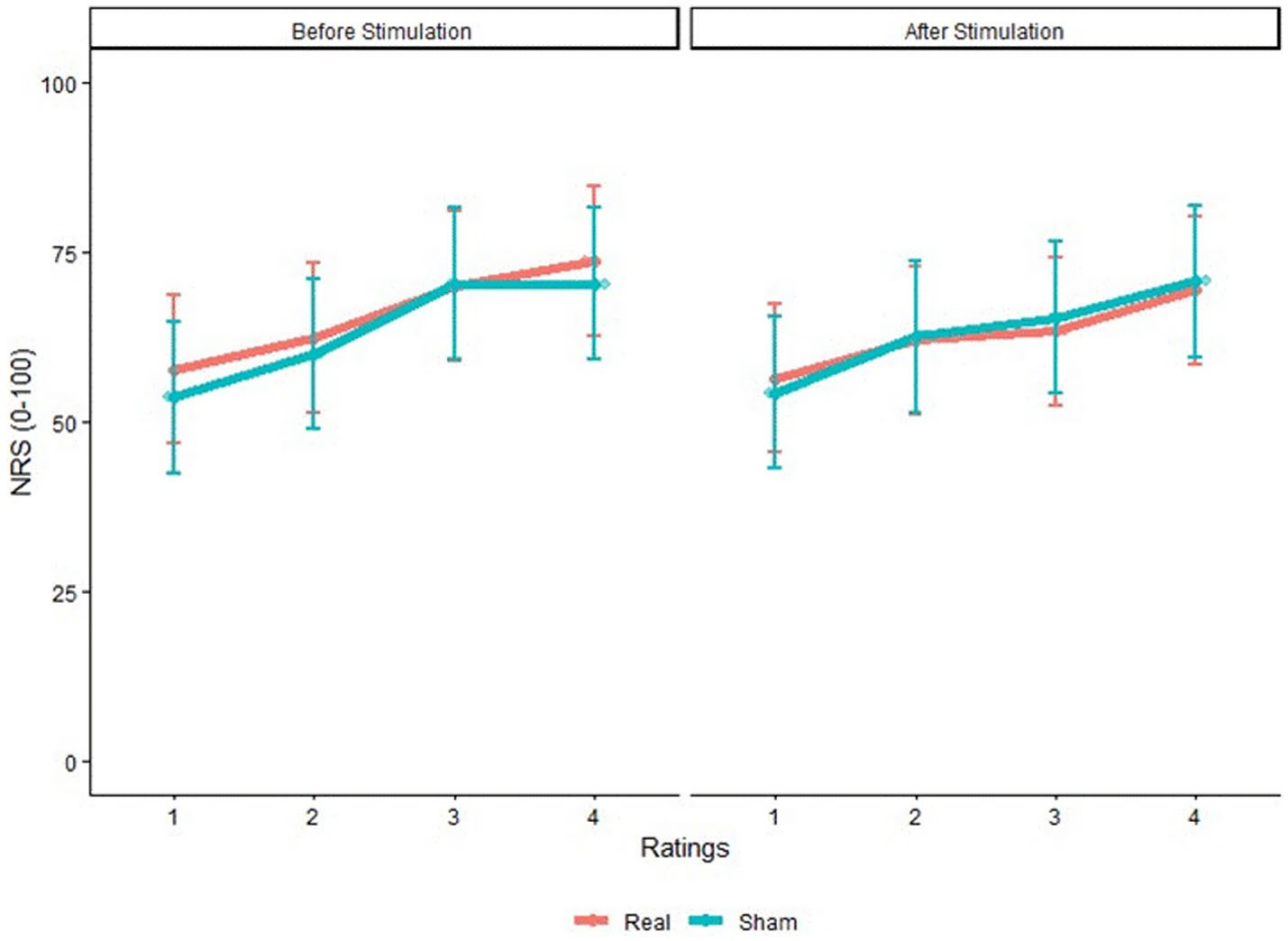
Pain intensity during tonic heat pain, before and after stimulation, as a function of the stimulation type (real, sham). Pain intensity in the real and sham tACS during the 120 s (x axis 1–30 s; 2–60 s, 3–90 s, and 4-120 s) of the tonic heat stimulation, before and after stimulation. NRS, Numerical Rating Scale.

**Figure 5:**
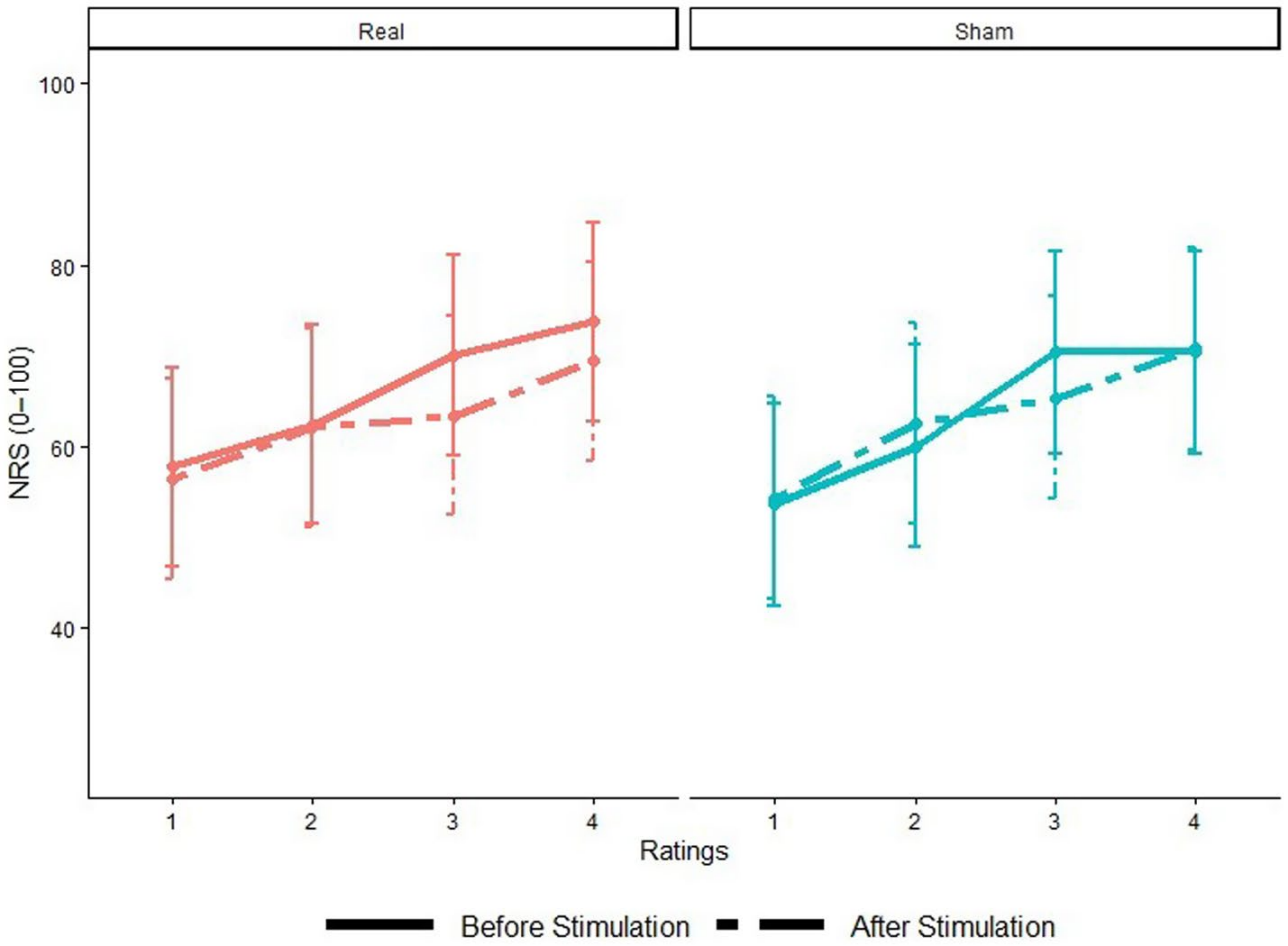
Pain intensity during tonic heat pain, in the real and sham tACS sessions as a function of time (before and after stimulation). Pain intensity before and after stimulation during the 120 s (x axis 1–30 s; 2–60 s, 3–90 s, and 4–120 s) of the tonic heat stimulation, in the real and sham tACS. NRS, Numerical Rating Scale.

Pain intensity reports before and after real and sham tACS are summarized in Table 3. A mixed-design ANOVA revealed a significant main effect of ratings (30, 60, 90, and 120 s during the THP stimulation), [*F*(3,174) = 27.04, *p* < 0.001, *ηp*^2^ = 0.32], indicating a significant change in pain during THP (30, 60, 90, and 120 s) across the time (before and after the intervention) and intervention (real tACS, sham tACS) (Figures 4, 5). Post-hoc pairwise comparisons showed that pain ratings significantly increased from the first to the second stimulus (*p* = 0.007) and from the first to the third stimulus (*p* = 0.021). No significant effects were found for time, intervention, or intervention order, and no interactions were identified among time × intervention, time x ratings, intervention × ratings, or time × intervention × ratings (Table 4).

**Table 3:** Pain intensity reports before and after real and sham tACS.

	Average	SD	Minimum	Maximum
Real tACS stimulation				
*Pain intensity before stimulation*				
At 30 s	58.0	21.1	30.0	90.0
At 60 s	62.7	17.7	35.0	90.0
At 90 s	71.3	16.4	50.0	100.0
At 120 s	74.0	16.6	50.0	100.0
Average of the 4 scores	66.3	18.0	41.2	95.0
*Pain intensity after stimulation*				
At 30 s	56.7	18.3	30.0	90.0
At 60 s	62.3	17.7	25.0	90.0
At 90 s	63.7	17.9	20.0	90.0
At 120 s	69.7	18.2	20.0	100.0
Average of the 4 scores	63.1	18.1	23.8	92.5
Sham tACS stimulation				
*Pain intensity before stimulation*				
At 30 s	52.9	19.2	20.0	80.0
At 60 s	59.3	22.4	20.0	90.0
At 90 s	69.6	19.0	25.0	100.0
At 120 s	69.6	23.6	10.0	100.0
Average of the 4 scores	62.9	21.1	18.6	92.5
*Pain intensity after stimulation*				
At 30 s	53.6	21.3	20.0	90.0
At 60 s	61.8	23.6	15.0	90.0
At 90 s	64.6	21.3	10.0	85.0
At 120 s	70.0	18.8	20.0	100.0
Average of the 4 scores	62.5	21.3	16.3	97.5

SD, standard deviation.

**Table 4:** The effects of tACS (real vs. sham) on pain intensity.

	*F*-value	*p*-value	Effect size^a^
Intervention	0.09	0.76	6.69e−03
Time	1.21	0.28	0.04
Rating	27.04	<0.001	0.32
Intervention order	0.03	0.86	2.13e−03
Intervention × time	0.76	0.38	0.03
Intervention × ratings	0.44	0.72	7.62e−03
Time × rating	1.31	0.27	0.02
Intervention × time × rating	0.08	0.77	1.14e−03

a Partial *η*^2^, effect size, according to Cohen (1988) considered 0.01 partial eta squared effect as small, 0.06 considered medium and 0.14 considered large.

### The relation between baseline pain intensity and PAF

A mixed model was fitted to investigate the effect of PAF at C3 and C4 on pain ratings at baseline. Results indicated a significant negative association between PAF at C3 and pain intensity [*β* = −9.18, SE = 4.45, *t*(45) = −2.06, *p* = 0.045]. The relationship between PAF at C4 and pain intensity was also negative but not statistically significant [*β* = −8.06, SE = 5.72, *t*(45) = −1.41, *p* = 0.16].

### Blinding efficacy

Twelve (80%) of the participants correctly guessed the type of tACS (real vs. sham) applied. Two participants (13.3%) incorrectly identified the stimulation type, and 1 participant could not decide about the stimulation type. No differences were found between stimulation sequence in blinding efficiency (*p* = 0.27).

## Discussion

This double-blind, randomized controlled study tested whether tACS delivered at 1 Hz above the iPAF can shift offline neural PAF and modulate experimental pain sensitivity (aftereffects) in healthy individuals. The main findings are that, within the limits of this preliminary study, we did not detect offline after-effects of tACS on iPAF or experimental pain sensitivity compared with the sham condition. Nevertheless, we replicate the findings of previous studies that continuous experimental pain shifts the iPAF toward low frequencies (Ploner et al., 2006; Peng et al., 2014; Nickel et al., 2020), and a faster iPAF is associated with reduced sensitivity to continuous experimental pain (Furman et al., 2018, 2019, 2020).

tACS has the potential to induce neural plasticity by modulating the brain’s neural oscillations at a specific frequency, to be synchronized with the external stimulus frequency (Hohn et al., 2019; Polanía et al., 2018; Tavakoli and Yun, 2017; Krause et al., 2019; Liu et al., 2018). This Non-Invasive Brain Stimulation (NIBS) technology enables testing the causal nature of oscillation-behavior relationships. Previous studies investigating the links between pain and neural oscillations have revealed associations between PAF amplitude and experimental pain intensity (Ploner et al., 2017; Peng et al., 2014; Furman et al., 2018). Therefore, tACS has been focused on facilitating alpha neuronal activity to reduce experimental or clinical pain (Fathi et al., 2026; May et al., 2021; Fathi et al., 2025). In these studies, tACS was applied at a fixed frequency of 10 Hz, which falls within the middle range of the alpha frequency range (8–12 Hz). In the current study, we applied tACS at 1 Hz over the iPAF for the first time, aiming to shift the PAF to a faster frequency and, thereby, reduce pain sensitivity to prolong experimental pain. The nonsignificant interactions we found suggest that the real and sham tACS did not differ reliably in their pattern of change across conditions (Resting state; After 1st pain stimulation; After tACS; After 2nd pain stimulation). The sensitivity analysis indicated that the study had sufficient sensitivity to detect small-to-medium interaction effects, suggesting that the findings do not support a robust intervention-related effect under the current stimulation parameters. However, smaller effects may have gone undetected. Therefore, the absence of a significant interaction should not be interpreted as definitive evidence for the absence of any tACS effect. Consistent with this, we also found no effect of tACS on pain rating and pain temporal summation. The latter may be because temporal summation reflects dynamic central facilitation of nociceptive signals, particularly at the spinal level, whereas tACS may modulate ongoing cortical alpha activity. Given the pilot nature of the study, these findings should be considered preliminary and useful for refining hypotheses, optimizing stimulation parameters, and informing the design of future confirmatory studies.

We did not record EEG during tACS stimulation because tACS artifacts are difficult to filter. Therefore, we cannot assess the physiological effects during application of tACS. Nevertheless, Vossen et al. (2015) findings suggest that aftereffects of tACS applied at ± 2 Hz relative to the individual alpha frequency do not exhibit any of the characteristics of entrainment echoes (Polanía et al., 2018; Vosskuhl et al., 2018; Tavakoli and Yun, 2017; Helfrich et al., 2014), and indicate that online tACS-entrainment effects may not be strong enough to outlast stimulation. In line, Vossen et al. (2015) found that alpha enhancement was not associated with a better match between the tACS frequency and the individual alpha frequency. Instead, greater enhancement was linked to greater deviation between the two. They and others (Vossen et al., 2015; Zaehle et al., 2010; Wischnewski et al., 2019) suggested that tACS-induced plasticity is based on spike-timing-dependent plasticity (STDP) rather than entrainment; tACS can shift spike timing so that pre- and postsynaptic activity repeatedly falls within timing windows that favor either long-term potentiation or depression. This model matched Vossen et al. (2015) data, showing that slower stimulation relative to the individual alpha frequency enhanced oscillations at the individual alpha frequency (i.e., faster). In this case, presynaptic events slightly precede postsynaptic events of the next cycle, leading to synapse strengthening (long-term potentiation). Taken together, a better understanding of the neurophysiological mechanisms underlying tACS-induced plasticity and related behavioral effects (here, pain sensitivity) can help inform research design choices and stimulation parameters.

The question of whether tACS can modulate PAF using different tACS protocols was investigated in a recently published meta-analysis. This includes frequency-specific stimulation above or below the individual PAF (+1 Hz, +2 Hz, −2 Hz) and non-individualized fixed-frequency or closed-loop approaches that dynamically adjust tACS based on recalculated PAF (Millard et al., 2024). Despite this methodological variability, the meta-analysis revealed no significant overall effect of tACS on PAF. Further analysis of specific protocols showed that stimulation above individual PAF (+1 Hz or +2 Hz) (Pahor and Jaušovec, 2016; Ronconi et al., 2022) did not result in significant increases in PAF, nor did stimulation below PAF (−2 Hz) (Ronconi et al., 2022) lead to substantial decreases. Collectively, the results of the meta-analysis suggest that a single tACS session across a range of commonly used parameters does not modulate PAF. This raises important questions about tACS’s capacity to induce frequency-specific neuromodulatory effects under current experimental conditions.

Evidence suggests that tACS may influence oscillatory dynamics beyond the targeted frequency band via several neurophysiological mechanisms, including cross-frequency coupling. Due to the brain’s interconnected network architecture, stimulation at one frequency and cortical site can propagate to functionally connected regions, potentially entraining oscillations such as theta or beta (Orendáčová and Kvašňák, 2025; Wischnewski et al., 2023). We did not investigate the intervention’s effects on broader oscillatory bands and networks, as this was not the aim of our study. However, the effect size on behavioral outcomes (i.e., pain sensitivity) was very small, further supporting the lack of changes in resting-state EEG that persist beyond the stimulation duration.

The literature suggests that brain oscillations during tonic noxious stimulation differ from those during brief noxious stimuli. During continuous pain, there is a decrease in alpha oscillations in sensorimotor areas, as measured by contralateral-central electrodes, and an increase in gamma oscillation power in the medial prefrontal (Peng et al., 2014; Schulz et al., 2015; Nickel et al., 2017). Moreover, these studies suggest that gamma oscillations encode subjective pain intensity and that objective stimulus intensity is negatively correlated with alpha oscillations. Thus, while studies successfully altered visually or auditory functions following a single session of tACS applied above or below the individual peak brain frequencies (Baltus et al., 2018; Cecere et al., 2015; Mioni et al., 2020), perhaps to modify pain sensitivity, a multi-session tACS is required.

A few limitations warrant consideration: First, this was a pilot study with a small sample size, and the results should be viewed as a hypothesis-generating step. Nevertheless, the findings may be useful in guiding the design of future studies examining tACS effects on pain. Second, although the goal was to deliver stimuli at 1 Hz above the iPAF, after the first pain stimulation, the iPAF shifted toward a lower frequency; therefore, in most cases, the stimulation frequency was slightly more than 1 Hz above the iPAF. Yet, Vossen et al.’s (2015) result suggests that a match between stimulation frequency and the endogenous frequency, i.e., iPAF, may not be required to produce tACS aftereffects. Nevertheless, future studies are suggested to reassess iPAF immediately before stimulation. Further, because the present study was designed to examine offline after-effects, EEG was recorded before and after stimulation, but not during tACS. Therefore, the findings should be interpreted as indicating no detectable offline modulation of iPAF or pain sensitivity under the current parameters. Future studies using online EEG-tACS recordings are needed to clarify the immediate physiological effects of stimulation and their relationship to post-stimulation outcomes.

Finally, based on patients reports, the blinding approach was not ideal, as most participants could guess which was the active treatment. This suggests that even the low 1 mA stimulation intensity generated sensations on the skin that were perceived differently than those experienced in the sham stimulation. In similar studies using the same stimulation parameters, the quality of blinding was either not reported (Antal et al., 2020; Lin et al., 2022) or successful (Ahn et al., 2019; Prim et al., 2019). However, in Prim et al. (2019), the assessment of blinding was conducted by asking participants to rate on a 0–100 VAS how confident they were that they received the stimulation, while we asked them to guess which session involved the real treatment (a binary question). It might be that when forced to choose, most participants are able to identify real vs. sham stimulation. To better understand this point, we recommend assessing the quality of blinding in both ways in future tACS studies. Although blinding was unsuccessful in the present study, the estimated intervention effect on PAF was negligible. Given that expectancy effects would more plausibly bias results toward detecting differences between active and sham stimulation, the absence of a measurable effect suggests that PAF was not meaningfully modulated under the current stimulation parameters.

## Conclusion

In this double-blind, randomized, controlled study, tACS applied at 1 Hz above iPAF did not induce offline changes in iPAF nor decrease experimental pain sensitivity compared to sham, despite replicating the expected pain-related slowing of iPAF and its link to pain sensitivity. Given the negligible intervention effect, the findings suggest that this frequency-tailored, single-session protocol is unlikely to modulate alpha peak dynamics. More broadly, they align with meta-analytic evidence that commonly used tACS parameters do not consistently modulate PAF, possibly because tACS effects are not necessarily based on entrainment but rather on spike-timing-dependent plasticity (Vogeti et al., 2022).

## Data Availability

The raw data supporting the conclusions of this article will be made available by the authors, without undue reservation.
